# Changes in Income after an Industrial Accident According to Industry and Return-to-Work Status

**DOI:** 10.3390/ijerph16142603

**Published:** 2019-07-22

**Authors:** Suk Won Bae, Sarah Soyeon Oh, Wha Me Park, Jaehoon Roh, Jong-Uk Won

**Affiliations:** 1 Department of Public Health, Graduate School, Yonsei University, Seoul 03722, Korea; 2 The Institute for Occupational Health, Yonsei University College of Medicine, Seoul 03722, Korea; 3 Institute of Health Services Research, Yonsei University College of Medicine, Seoul 03722, Korea; 4 Graduate School of Public Health, Yonsei University, Seoul 03722, Korea; 5 Incheon Workers’ Health Center, Incheon 21633, Korea; 6 Department of Preventive Medicine, Yonsei University College of Medicine, Seoul 03722, Korea

**Keywords:** industrial accident, industry, return-to-work status, workers’ compensation insurance

## Abstract

*Objective:* To investigate changes in the incomes of workers, particularly those in the construction sector, who experienced industrial accidents according to their status of return to work. *Methods:* We used data from the fifth Panel Study of Workers’ Compensation Insurance. A repeated measures ANOVA was used to compare annual differential incomes before and after the industrial accident, and a linear mixed model was used to investigate the changes in income from before to after the industrial accident according to the industry and return-to-work status. *Results:* A comparison of the industrial categories revealed that construction industry workers exhibited the greatest incomes before the accident and the greatest decrease in income after the industrial accident. Regression analysis for assessing changes in income after the industrial accident showed that a comparison by industry revealed a significantly greater reduction in income in the construction than service industry. A comparison by work status revealed significantly greater decreases in income in the reemployment and non-return to work groups than among those who returned to their original work. *Conclusions:* The economic statuses of the victims of industrial accidents decreased relative to the pre-accident statuses in all industries. The ability to return to original work is important for preserving the accident victim’s economic status.

## 1. Introduction

Although the number of Korean workers involved in industrial accidents has declined steadily from 2008 to 2017, the annual number of reported cases continues to exceed 90,000 [1,2,3]. Industrial accidents have various negative consequences, including high social costs and loss of productivity in the workplace [3,4,5,6]. The injured workers also face issues associated with recovery and loss of income [7,8,9]. Furthermore, the loss of labor associated with industrial accidents can be considered a national economic problem rather than an individual one [10,11]. In Korea, when an occupationally injured worker who has completed medical care still has a remaining mental or physical disability and is eligible for disability benefit payments, the benefits are calculated by multiplying the number of days commensurate with the disability grade by the average wage and then paid to the worker [1]. Grades 1–3 are payable only as pension and can be paid in lump sum of half a year to 4 years. Grades 4–7 are offered as payment options of lump sum or monthly pension payments. If pension is chosen, half of 2 years’ worth of pension can be received in lump sum. Grades 8–14 can only be paid in lump sum [3].

The direct loss (industrial compensation pay-out amount) from industrial accidents in 2016 in Korea was about 4 trillion won (3.4 billion USD), and the estimated economic loss including indirect loss was about 21 trillion won (17.7 billion USD) [3]. In 2017, the economic losses attributed to industrial accidents totaled approximately 22 trillion won (18.6 billion USD), an increase of 3.64% relative to the previous year. Moreover, 47 million lost work days associated with accidents were reported in 2017, which is an increase of 0.68% from 2016. Of the victims, 28.20% and 28.55% were employed in the manufacturing and construction sectors, respectively [1]. Although the latter sector contributes to national economic growth and provides numerous employment opportunities [4,12], it is associated with high rates of overall industrial accidents and severe and fatal accidents [13,14]. The Korean Workers’ Compensation and Welfare Service (KCOMWEL) provides various types of support to people with occupational injury or illness, including monetary compensation, rehabilitation services [6]. Additionally, the KCOMWEL focused on return-to-work (RTW) for occupationally injured workers and developed various programs to promote RTW [2]. A return-to- work after an industrial accident has been identified as the most effective way to reduce personal or national losses [15,16]. However, the majority of construction workers are employed on a daily basis and find it difficult to return-to-work and receive services after an accident.

Several studies have identified the factors affecting a worker’s ability to resume their occupation. Briefly, a return-to-work is easier for workers who are male, younger, and have better health, a low level of disability from injury, a shorter recovery period, better hospital quality and a greater interest in returning to work from the physician [2,17,18,19,20,21,22,23]. Some additional studies evaluated compensation for industrial accidents or the return-to-work and changes in income after industrial accidents [3,10,24,25,26]. However, those studies were based on either the degree of disability after the accident or the status of workers before the accident. In contrast, few studies focused directly on the changes in income after the accident from the industrial perspective or on the status of return-to-work.

The purpose of this study was to examine (1) how the income of industrial workers changes depending on the type of industry they were employed in before industrial accidents; (2) how the income of industrial workers changes after return to one’s job after an accident; and (3) the differences between income before the accident and five-year average earnings after the accident.

## 2. Materials and Methods

### 2.1. Study Design and Participants

For this study, we used data from the fifth Panel Study of Workers’ Compensation Insurance (PSWCI), which was conducted by the Korean Workers’ Compensation and Welfare Service. This survey targeted 82,493 workers who experienced work-related injuries for which medical care was terminated in the year 2012. The final sample population of 2000 workers was selected proportionally from nine regions after the priority assignment of disability grades across six levels. The first panel study was conducted in 2013 and has since been conducted annually. Accordingly, the fifth study obtained survey data during 2017, using a computer-assisted personal interviewing (CAPI) method together with face-to-face interviews conducted by skilled interviewers [2,3,22,23,27,28,29,30]. The fifth survey included 1616 workers who had participated since the first survey, yielding a retention rate of 80%. However, only 1514 workers had participated continuously during the five-year period. We excluded 41 workers who had not participated in questionnaire item or whose explanatory variable was difficult to estimate. On the contrary, our previous study, which used data from the fourth PSWCI survey, used a sample of 1660, and 1588 of them continued to participate in the fourth PSWCI. A total of 50 participants were excluded as their data were incomplete [3]. We also excluded an additional 345 workers, who were not excluded in our previous study but were categorized as working in other industries (agriculture, forestry, fishing, etc.) because we only examined workers in manufacturing, construction, and service industries; our final sample for analysis included 1128 workers.

### 2.2. Sociodemographic Characteristics

Participants were classified by age into five groups: <30, 30–39, 40–49, 50–59 and ≥60 years. The marital status was classified into three categories: Not married, married and others (e.g., separated, divorced, widowed). The educational level was also classified into three groups: Less than high school, high school and college or above.

### 2.3. Occupational Characteristics

We followed the Korean Standard Industrial Classification (KSCI) guideline, which is based on the International Classification of Standard Industry (ISCI), to categorize industrial classifications. In 2016, more than half of all work-related injuries and illnesses occurred in the manufacturing (28.8%) and construction sectors (29.3%). Therefore, we divided industrial classifications into three categories: Manufacturing, construction and service. Participants were further categorized as regular workers and daily workers (including temporary workers).

The Korean Industrial Accident Compensation Insurance Act uses 14 grades to rate the level of disability. Using this scale, a lower grade represents more severe disability [31]. For our study, we used these data to classify injury cases into five categories: 1–3 (critical), 4–7 (severe), 8–10 (moderate), 11–14 (mild) and none.

The industrial accidents were classified into injury and disease. The number of employees in the workplace at the time of industrial accidents was classified into four groups: <5, 5–9, 10–29, and ≥30. The duration of employment at the workplace where workers experienced industrial accidents was classified into three groups: <1 year, 1–less than 3 years, and ≥3 years.

The PSWCI survey used six categories to describe a return-to-work, including a return to original work, reemployment, self-employment, unpaid family worker, unemployment and non-economic activities. We recategorized the options into return to original work, which included those who returned to the work performed prior to the accident; reemployment, which included those employed by a different employer; and non-return to work, which included participants who were self-employed, unpaid family workers, unemployed and those participating in non-economic activities.

### 2.4. Main Outcome Variables

Our main outcome variables are incomes pre- and post-industrial accidents. Income before the industrial accident was estimated using the question “What is the average monthly wage at the workplace (i.e., job) where the industrial accident occurred?”. For a period of medical treatment that lasted between 1 and 2 years, with the assumption that 2011 was the year of the incident, the income data were approximated by multiplying the average monthly wage with the rate of change that was stated for the year 2012 by the Korean Ministry of Employment and Labor. For a treatment period that exceeded 2 years, the corresponding data were omitted as approximation was impossible [3]. The post-accident income was calculated for each participant as the sum of the earned income and non-work income. The earned income included wage and business income, while non-work income included industry-related income. Sources of income not related to the industrial accident (e.g., property and private income) were excluded. To compare the average incomes from before and after industrial accident, we used the annual rate of income increase to adjust all wages to the 2016 standards [3].

### 2.5. Statistical Analyses

We used the *t*-test and analysis of variance (ANOVA) to compare the general characteristics of the study population and the average incomes before industrial accidents according to the industrial classification (manufacturing, construction, or service). A repeated measures ANOVA was used to compare annual differential incomes before and after the industrial accident, as well as the annual income before the industrial accident and the average annual income 5 years after the accident. A paired *t*-test and repeated measures ANOVA were conducted to compare the income before the accident and the average annual income 5 years later according to the general characteristics of the study population. To investigate the changes in income from before to after the industrial accident according to industry and RTW status, we set the dependent variable as the income minus the income before the accident from the five-year average income and performed a linear mixed model analysis. All statistical analyses were conducted using the SAS statistical package, version 9.4 (SAS Institute, Cary, NC, USA).

## 3. Results

The general characteristics of participants and incomes prior to the industrial accidents are shown in Table 1 according to the industrial classification. The average incomes before the industrial accident were 27.36, 31.48 and 22.40 million KRW (23,000, 27,000 and 19,000 USD) in the manufacturing, construction and service industries, respectively. The service industry included a relatively higher proportion of female workers (44.6%) and a significantly lower pre-accident income (*p* < 0.0001). The construction industry included large proportions of daily workers and workers who had worked for <1 year. However, no differences in income before the accident were observed with respect to status of workers or work duration (*p* = 0.4100 and *p* = 0.1430, respectively), whereas increases in income with longer working periods were observed in both the manufacturing and service industries. The construction industry had a lower rate of return to original work, compared to the manufacturing and service industries, although the income levels of those who returned to original work and those who were reemployed were similar (*p* = 0.0311).

A comparison of the industrial categories revealed that the construction industry workers exhibited the greatest incomes before the accident and the greatest decrease income after the industrial accident (*p* < 0.0001) (Table 2). The income of the first year after the workers’ industrial accident medical treatment was higher than that prior to the accident for all workers. This was followed by a dramatic decline in the second year and increases in subsequent years. For all workers, the five-year average incomes were lower than the income priors to the accident (*p* < 0.0001).

Table 3 presents the differences between the income prior to the industrial accident and the five-year average income after the accident. This analysis revealed significant decreases in income after the accident (*p* < 0.0001). The five-year average incomes after the accident were lower than the income prior to the accident for all workers except those aged 30–39 years, who returned to their original workplace, and with ≥3 years of work experience.

As per the characteristics of each year by the industrial classification and return-to-work status, the proportion of workers who returned to their original work, as well as their incomes, tended to decrease after the first year (Table 4). Particularly, workers in the construction industry had significantly lower rates of return to original work, 8.9–13.9%, compared to the manufacturing (35.1–46.9%) and service industries (37.1–47.3%). However, the first-year incomes after the accidents did not differ among workers who returned to original work, were reemployed and did not return to work (*p* = 0.0568).

Finally, the changes in income after the industrial accident were subjected to a regression analysis in which the dependent variable was the five-year average income after the industrial accident, minus the income prior to the accident (Table 5). The independent variables were the workers’ general characteristics, industrial classification, return-to-work status and other variables. After adjusting all covariates, the analysis revealed a significantly greater reduction in income for male worker, compared to female workers (*p* = 0.0069). A comparison by industry revealed a significantly greater reduction in income in construction, compared to service industry (*p* = 0.0010). A comparison by work status revealed significantly greater decreases in income in the reemployment and non-return to work groups, compared to those who returned to original work (*p* < 0.0001 for both).

## 4. Discussion

This study aimed to investigate and compare the industrial accident victims’ incomes before and after the accidents, RTW, and differences in these incomes according to the industry and RTW status.

In our study, we found that construction workers had the highest pre-accident incomes (Table 1). In 2016, the Korean Ministry of Employment and Labor reported total wage expenditures of 45.06, 32.08 and 24.92–57.80 million KWR (38,000, 27,000 and 21,000–49,000 USD) for the manufacturing, construction and service industries, respectively [32]. A comparison with those data suggests that the panel survey data used in this study overestimated the pre-accident income in the construction industry. Moreover, the victims of industrial accidents in the manufacturing sector may have been production workers with an income lower than the total average income in this sector. Especially, Table 1 shows the characteristics of construction industry that are different from the manufacturing and/or service industries. The characteristics of the pre-accident income are the same as those of the general employees’ [3]. As seen in the existing results, the construction industry had a high percentage of daily workers [33,34], who had an undetermined number of work days and received wages daily. In addition, because there was neither wage differential between regular and daily workers, nor differences among those with varying durations of employment, there may not have been differences in income before the industrial accident. Results of industrial workers in the construction sector, who had a short duration of employment, was consistent with the findings from a previous study [35]. According to data published by the Korean Ministry of Employment and Labor, the industrial accident percentage of workers who had a duration of employment of less than 1 year was 95.1% in construction, 45.8% in manufacturing, and 46.1% in service sectors [1]. We also obtained similar results (Table 1), and therefore, our results can be considered to represent Korean industries.

We further found that the first-year income of workers in all three industries increased from the income prior to the accident, decreased dramatically in the second year, and increased again thereafter (Table 2). This trend was similar to a previous study, which found that an increased income during the first year after the disaster could be attributed to lump-sum payments of disability benefits [3]. Although the construction industry reported the highest total income prior to the industrial accident (36.41 vs. 31.65 and 25.91 million KWR—27,000 vs. 31,000 and 22,000 USD for manufacturing and service sectors, respectively), it also exhibited the greatest decrease compared to the five-year average income after industrial accidents. Income before the industrial accident was estimated using the question “What is the average monthly wage at the workplace (i.e., job) where the industrial accident occurred?” [3]. The income before the industrial accident may not have been measured accurately because the survey method was based on personal statements. The probability of overestimating the income before the industrial accident among construction workers is especially high for daily workers [33,34]. If daily workers, who have an undetermined number of work days and receive wages daily, reported pre-accident average income that included weekends and holidays, it is possible that a higher amount than the actual income is recorded in the Panel Study of Workers’ Compensation Insurance. The Korean Industrial Accident Compensation Insurance Act states that insurance and related benefits are based on a worker’s average wage. For daily workers, however, these benefits are calculated by multiplying the daily wage by a labor coefficient of 0.73 [31].

Our analysis revealed that among workers in all three industries the rate of return to the original workplace decreased after the first year of treatment, while the reemployment rate tended to increase simultaneously (Table 4). Workers who returned to the original workplace reported a higher five-year average income after the industrial accident than the pre-accident income (Table 3). As the number of years progressed, income decreased as the percentage of those who returned to the original workplace reduced. The low rate of construction workers returning to their original workplace after an industrial accident is also consistent with the results of previous research. Because injuries in the construction industry are typically more serious than those in the manufacturing and service industries [35], income is inevitably reduced. In a previous study, clear and significant differences in income were observed between employed and unemployed workers, with lower values in the latter group [3]. In our study, however, we found no wage gap between regular and daily construction workers and there was no difference in wage by continuously provided service year (Table 1) that the wage gap between return to original work and reemployment was small.

According to data published by the Korean Ministry of Employment and Labor, the number of construction workers who experienced industrial accidents in 2016 was 26,000 [1] and that of those included in this study was 381 (Table 1). Nevertheless, this study’s data were obtained from a large-scale survey conducted by the KCOMWEL with workers who stopped receiving medical care in 2012 after experiencing industrial accidents [23,28,29,30], and as we systematically sampled this large-scale survey’s panel, our results can be considered to represent the population of such workers in Korea [2].

## 5. Conclusions

In conclusion, the economic statuses of the victims of industrial accidents decreased relative to the pre-accident statuses in all industries. However, this effect was particularly noticeable in the construction industry. Construction workers were also less likely than manufacturing and service workers to resume their original work. We additionally identified some differences in income between participants who returned to their original work and were reemployed in other fields and between regular and daily workers. Therefore, the ability to return to original work is important for the maintenance of the accident victim’s economic status. Furthermore, our findings emphasize the importance of efforts intended to encourage construction workers to return to original work or seek reemployment in other workplaces.

## Figures and Tables

**Table 1:** General characteristics of study subjects by industry before industrial accident (unit: million KRW).

Variables	Industry before Industrial Accident														
	Manufacturing				*p*-Value *	Construction				*p*-Value *	Service				*p*-Value *
	*N*	%	Mean	SD		*N*	%	Mean	SD		*N*	%	Mean	SD	
Total	561	100	27.36	12.60		381	100	31.48	11.44		186	100	22.40	13.39	
Age					<0.0001					0.0002					<0.0001
<30	38	6.8	22.51	7.07		6	1.6	21.14	4.44		14	7.5	25.52	11.56	
30–39	99	17.7	28.41	10.93		28	7.4	33.77	14.18		33	17.7	27.35	12.14	
40–49	160	28.5	30.06	13.84		92	24.2	31.59	11.32		35	18.8	30.89	17.89	
50–59	202	36.0	27.08	12.98		161	42.3	33.55	10.85		58	31.2	19.76	11.37	
≥60	62	11.1	22.61	11.06		94	24.7	27.79	10.83		46	24.7	14.75	6.52	
Sex					<0.0001					0.0002					<0.0001
Male	471	84.0	29.57	12.41		372	97.6	31.87	11.22		103	55.4	28.63	14.17	
Female	90	16.0	15.76	4.87		9	2.4	15.09	8.06		83	44.6	14.66	6.74	
Marital status					<0.0001					0.0988					<0.0001
Not married	91	16.2	23.07	6.84		37	9.7	28.90	10.70		31	16.7	25.12	9.46	
Married	411	73.3	28.96	13.62		279	73.2	32.19	11.62		132	71.0	23.05	14.64	
Others	59	10.5	22.84	9.05		65	17.1	29.87	10.81		23	12.4	14.95	6.22	
Education level					<0.0001					0.1353					<0.0001
Less than high school	180	32.1	22.90	9.83		209	54.9	30.51	11.17		65	35.0	15.21	8.23	
High school	303	54.0	28.96	12.73		142	37.3	32.89	12.02		62	33.3	23.40	12.50	
College or above	78	13.9	31.42	14.87		30	7.9	31.51	10.02		59	31.7	29.25	15.05	
Status of workers					0.0181					0.4100					<0.0001
Regular worker	453	80.8	27.99	12.48		47	12.3	30.18	11.47		112	60.2	25.79	14.56	
Daily worker	108	19.3	24.73	12.81		334	87.7	31.66	11.44		74	39.8	17.25	9.36	
Accident type					0.0065					0.7238					<0.0001
Injury	496	88.4	26.78	12.37		371	97.4	31.44	11.44		165	88.7	21.98	13.05	
Disease	65	11.6	31.74	13.59		10	2.6	32.82	11.85		21	11.3	25.67	15.80	
Number of employees					<0.0001					0.0588					<0.0001
<5	89	15.9	23.05	8.52		115	30.2	30.75	12.46		42	22.6	18.40	8.30	
5–9	111	19.8	25.01	12.07		121	31.8	31.67	11.15		38	20.4	19.66	8.82	
10–29	154	27.5	25.92	10.55		99	26.0	30.28	10.45		56	30.1	23.14	13.88	
≥30	207	36.9	31.54	14.50		46	12.1	35.35	11.06		50	26.9	27.00	17.40	
Duration of employment					<0.0001					0.1430					<0.0001
<1 year	278	49.6	24.33	10.58		361	94.8	31.73	11.57		88	47.3	18.77	9.61	
1–less than 3 years	107	19.1	25.26	11.10		11	2.9	26.11	8.15		36	19.4	17.61	8.40	
≥3 years	176	31.4	33.42	14.22		9	2.4	27.73	6.83		62	33.3	30.32	16.60	
Disability rating					0.4899					0.4222					<0.0001
1–3	2	0.4	23.25	2.34		2	0.5	28.02	13.21		.	.	.	.	
4–7	33	5.9	27.08	14.59		14	3.7	35.71	11.10		.	.	.	.	
8–10	107	19.1	25.93	10.86		82	21.5	32.22	12.27		20	10.8	23.69	12.79	
11–14	345	61.5	27.81	12.33		233	61.2	31.35	11.61		113	60.8	23.54	14.92	
None	74	13.2	27.54	15.24		50	13.1	29.81	9.05		53	28.5	19.46	9.30	
Return to work					<0.0001					0.0311					<0.0001
Return to original work	263	46.9	30.40	13.16		53	13.9	32.17	9.97		88	47.3	27.46	15.70	
Reemployment	135	24.1	25.62	10.89		186	48.8	32.73	11.89		59	31.7	18.96	9.18	
Non return to work	163	29.1	23.89	11.88		142	37.3	29.57	11.15		39	21.0	16.17	7.94	

* Analyses were done by using *t*-test, ANOVA, SD: Standard Deviation.

**Table 2:** Relationship between industry and annual income changes before and after industrial accident (unit: million KRW).

Variables	Income before Industrial Accident (2012) *		First (2012) *		Second (2013) *		Third (2014) *		Fourth (2015) *		Fifth (2016)		*p*-Value ^‡^	First to Fifth (2012–2016) ^†^		*p*-Value ^§^
	Mean	SD	Mean	SD	Mean	SD	Mean	SD	Mean	SD	Mean	SD		Mean	SD	
Industry													<0.0001			<0.0001
Manufacturing (*N* = 561)	31.65	14.58	39.22	24.67	27.88	20.62	26.39	17.86	27.62	16.88	27.38	19.87		29.70	16.78	
Construction (*N* = 381)	36.41	13.23	40.74	22.16	22.37	16.65	22.25	15.78	23.40	14.56	23.16	15.90		26.39	12.67	
Service (*N* = 186)	25.91	15.49	29.61	20.25	23.71	20.47	22.88	18.03	22.49	16.48	22.56	17.36		24.25	16.71	

* All adjusted to fit the wages of 2016. ^†^ Average of the first to fifth-year income. ^‡^ Analyses were done by using repeated measures ANOVA. ^§^ Analyses were done annual income before industrial accident and the average of the first to fifth-year annual income by using repeated measures ANOVA.

**Table 3:** Comparison of the income before industrial accident and the five-year average income after the industrial accident according to general characteristics (unit: million KRW).

Variables	Income before Industrial Accident *		Average of the First to Fifth-Year Income *		Difference	*p*-Value ^†^
	Mean	SD	Mean	SD	%	
Annual income	32.31	14.72	27.68	15.63	−14%	<0.0001 ^‡^
Age						<0.0001
<30	25.61	11.90	22.07	15.80	−14%	
30–39	29.94	11.72	30.64	14.81	2%	
40–49	35.78	14.52	33.01	15.89	−8%	
50–59	33.60	15.77	29.43	16.00	−12%	
≥60	29.79	14.02	21.85	13.32	−27%	
Sex						<0.0001
Male	35.14	14.13	30.18	15.50	−14%	
Female	17.61	6.88	14.70	8.05	−17%	
Marital status						<0.0001
Not married	28.75	10.12	24.55	11.09	−15%	
Married	33.62	15.30	29.56	16.70	−12%	
Others	28.99	14.09	21.35	10.75	−26%	
Education level						<0.0001
Less than high school	29.28	13.41	21.93	12.22	−25%	
High school	34.03	14.80	30.91	16.09	−9%	
College or above	35.21	16.38	33.29	17.46	−5%	
Industry						<0.0001
Manufacturing	31.65	14.58	29.70	16.78	−6%	
Construction	36.41	13.23	26.39	12.67	−28%	
Service	25.91	15.49	24.25	16.71	−6%	
Status of workers						<0.0001
Regular worker	32.11	14.86	30.98	16.87	−4%	
Daily worker	32.56	14.57	23.77	13.00	−27%	
Accident type						0.0004
Injury	32.03	14.55	27.06	15.04	−16%	
Disease	35.31	16.24	34.31	19.90	−3%	
Number of employees						<0.0001
<5	29.91	13.36	26.08	12.91	−13%	
5–9	31.52	13.91	24.30	12.19	−23%	
10–29	31.02	13.25	24.97	12.65	−20%	
≥30	36.29	17.01	34.76	20.27	−4%	
Duration of employment						<0.0001
<1 year	31.62	13.79	24.58	12.61	−22%	
1–less than 3 years	27.23	12.50	25.54	13.17	−6%	
≥3 years	37.52	17.01	38.16	20.01	2%	
Disability rating						0.0498
1–3	29.65	9.51	27.47	14.25	−7%	
4–7	34.30	16.31	32.60	14.91	−5%	
8–10	32.61	13.91	29.52	16.06	−9%	
11–14	32.74	14.82	27.38	15.88	−16%	
None	29.80	14.75	25.40	13.89	−15%	
Return to work						<0.0001
Return to original work	35.53	15.90	38.04	16.70	7%	
Reemployment	31.88	13.36	26.00	11.63	−18%	
Non return to work	29.56	15.47	19.49	15.64	−34%	

* All adjusted to fit the wages of 2016. ^†^ Analyses were done by using repeated measures ANOVA. ^‡^ Analyses were done by using paired *t*-test.

**Table 4:** Annual income characteristics between the industry and return to work (unit: million KRW).

Variables		First (2012) *				*p*-Value	Second (2013) *				*p*-Value	Third (2014) *				*p*-Value	Fourth (2015) *				*p*-Value	Fifth (2016)				*p*-Value
		*N*	%	Mean	SD		*N*	%	Mean	SD		*N*	%	Mean	SD		*N*	%	Mean	SD		*N*	%	Mean	SD	
Industry	Return to work																									
Manufacturing	Return to original work	263	46.9	47.63	26.14	<0.0001	239	42.6	38.18	19.21	<0.0001	228	40.6	36.49	16.52	<0.0001	209	37.3	36.53	16.11	<0.0001	197	35.1	36.66	15.53	<0.0001
	Reemployment	135	24.1	31.74	21.05		202	36.0	23.40	15.82		220	39.2	22.39	12.03		237	42.3	24.85	11.84		240	42.8	24.04	12.28	
	Non return to work	163	29.1	31.84	20.41		120	21.4	14.90	20.58		113	20.1	13.82	18.99		115	20.5	17.15	19.08		124	22.1	19.10	29.87	
Construction	Return to original work	53	13.9	46.13	20.82	0.0568	47	12.3	33.16	13.20	<0.0001	42	11.0	32.14	11.23	<0.0001	37	9.7	32.77	9.67	<0.0001	34	8.9	32.46	9.76	<0.0001
	Reemployment	186	48.8	38.33	23.14		238	62.5	25.26	15.75		238	62.5	26.44	14.20		247	64.8	27.41	12.40		248	65.1	27.10	14.31	
	Non return to work	142	37.3	41.90	21.01		96	25.2	9.93	13.07		101	26.5	8.27	11.65		97	25.5	9.60	11.93		99	26.0	10.11	13.79	
Service	Return to original work	88	47.3	37.63	22.35	<0.0001	85	45.7	33.61	21.69	<0.0001	74	39.8	34.04	19.60	<0.0001	72	38.7	32.58	17.87	<0.0001	69	37.1	33.33	18.24	<0.0001
	Reemployment	59	31.7	22.27	14.70		69	37.1	18.26	13.35		75	40.3	19.00	10.92		79	42.5	19.23	10.58		76	40.9	18.95	11.03	
	Non return to work	39	21.0	22.61	15.52		32	17.2	9.17	16.76		37	19.9	8.40	11.98		35	18.8	9.08	11.37		41	22.0	11.14	15.55	

* All adjusted to fit the wages of 2016.

**Table 5:** Relationship between the general characteristics and five-year average of post-accident income minus the income before the industrial accident (unit: million KRW).

Variables	ß *	SE	*p*-Value
Age			
<30	ref		
30–39	2.18	2.87	0.4482
40–49	−1.36	2.83	0.6318
50–59	−2.63	2.84	0.3546
≥60	−3.52	2.90	0.2258
Sex			
Male	−2.79	1.03	0.0069
Female	ref		
Marital status			
Not married	−2.22	1.17	0.0576
Married	ref		
Others	−1.13	0.95	0.2345
Education level			
Less than high school	−0.47	1.19	0.6931
High school	0.37	1.04	0.7239
College or above	ref		
Industry			
Manufacturing	−0.67	1.03	0.5165
Construction	−4.08	1.23	0.0010
Service	ref		
Status of workers			
Regular worker	ref		
Daily worker	−1.67	0.93	0.0709
Accident type			
Injury	−0.59	1.23	0.6302
Disease	ref		
Number of employees			
<5	1.77	0.99	0.0738
5–9	−1.21	0.97	0.2115
10–29	−1.29	0.92	0.1602
≥30	ref		
Duration of employment			
<1 year	−1.62	1.05	0.1226
1–less than 3 years	−0.56	1.19	0.6417
≥3 years	ref		
Disability rating			
1–3	ref		
4–7	−1.73	5.72	0.7621
8–10	−3.47	5.56	0.5323
11–14	−7.20	5.52	0.1923
None	−7.28	5.58	0.1926
Return to work			
Return to original work	ref		
Reemployment	−5.17	0.89	<0.0001
Non return to work	−10.04	1.02	<0.0001

* Statistical estimated from a general linear model that adjusted for all other covariates excluding an interesting variant, SE: Standard Error.
